# Development of a Compatible Taper Function and Stand-Level Merchantable Volume Model for Chinese Fir Plantations

**DOI:** 10.1371/journal.pone.0147610

**Published:** 2016-01-22

**Authors:** Xiaolu Tang, César Pérez-Cruzado, Lutz Fehrmann, Juan Gabriel Álvarez-González, Yuanchang Lu, Christoph Kleinn

**Affiliations:** 1 Chair of Forest Inventory and Remote Sensing, Georg-August-Universität Göttingen, Göttingen, Germany; 2 Departamento de Ingeniería Agroforestal, Universidad de Santiago de Compostela, Lugo, Spain; 3 Department of Forest Management and Statistics, Institute of Forest Resource Information techniques, Chinese Academy of Forestry, Beijing, PR of China; Pennsylvania State University, UNITED STATES

## Abstract

Chinese fir (*Cunninghamia lanceolata* [Lamb.] Hook) is one of the most important plantation tree species in China with good timber quality and fast growth. It covers an area of 8.54 million hectare, which corresponds to 21% of the total plantation area and 32% of total plantation volume in China. With the increasing market demand, an accurate estimation and prediction of merchantable volume at tree- and stand-level is becoming important for plantation owners. Although there are many studies on the total tree volume estimation from allometric models, these allometric models cannot predict tree- and stand-level merchantable volume at any merchantable height, and the stand-level merchantable volume model was not seen yet in Chinese fir plantations. This study aimed to develop (1) a compatible taper function for tree-level merchantable volume estimation, and (2) a stand-level merchantable volume model for Chinese fir plantations. This “taper function system” consisted in a taper function, a merchantable volume equation and a total tree volume equation. 46 Chinese fir trees were felled to develop the taper function in Shitai County, Anhui province, China. A second-order continuous autoregressive error structure corrected the inherent serial autocorrelation of different observations in one tree. The taper function and volume equations were fitted simultaneously after autocorrelation correction. The compatible taper function fitted well to our data and had very good performances in diameter and total tree volume prediction. The stand-level merchantable volume equation based on the ratio approach was developed using basal area, dominant height, quadratic mean diameter and top diameter (ranging from 0 to 30 cm) as independent variables. At last, a total stand-level volume table using stand basal area and dominant height as variables was proposed for local forest managers to simplify the stand volume estimation.

## Introduction

As an important plantation species, Chinese fir (*Cunninghamia lanceolata* [Lamb.] Hook) has been widely planted throughout Southern China for more than one thousand years history and is famous for its fast growth and good quality [1,2]. Currently, Chinese fir plantations have an area of 8.54 million ha, amounting for 21% of the China’s total plantation area and 32% of total plantation tree volume according to the 7^th^ national forest inventory [3]. With the changes of market and the increasingly local economic dependence, timber production has become the most important object in Chinese fir plantations not only for plantation owners, but also for local governments. Chinese fir plantations are usually managed in a clear cutting system with a rotation period of 20 to 30 years depending on the site quality [4]. Accurate prediction of volume in such rotation period is a matter of interest for plantation owners and forest managers.

To estimate the total tree volume, allometic models using diameter at breast height (1.3 m, *D*) and total tree height have been widely used [5–7]. However, these models cannot predict the volume to any height limits and merchantable volumes to any merchantable height or diameter limits [8]. Instead, taper functions have been well developed to solve such problems. Taper functions do not only describe the stem shapes [9], but also provide the estimation of: (1) diameter at any height of the stem; (2) total tree volume; (3) merchantable volume and merchantable height at any top diameter and (4) individual volumes for logs between any two heights [10,11].

Ideally, a taper function should be compatible. For example, the volume calculation from the estimation of taper function should be equal to that computed from a total volume model [12–14]. It is also desirable to achieve the compatibility that the merchantable volume to any merchantable height calculated from taper function should be equal to that from a merchantable volume model [11]. Many compatible taper functions have been proposed for different tree species with different forms, as reviewed by Diéguez-ArandaCastedo-DoradoÁlvarez-González and Rojo [15]. These taper functions are classified into three types: single taper models, segmented taper models and variable-form taper models. Through comparing different taper functions for different tree species, the segmented taper function developed by Fang et al. [14] is regarded as one of the most accurate and flexible functions that has been used for different tree species [8,11,15].

Some direct and indirect approaches have been developed to estimate the merchantable volume. Direct approaches measure the diameter at different heights and calculate the section volume up to the merchantable height or diameter of interest. Indirect approaches focus on (i) the development of a volume equation to a fixed merchantable diameter or merchantable height; (ii) the development of a taper function that is compatible to estimate the merchantable volume [11,16]; (iii) the development of a merchantable volume that is proportional to the total stem volume using a merchantable diameter or height [17,18]. Among these methods, volume-ratio equations that predict merchantable volume as a percentage of total volume and taper functions are the most widely used to estimate merchantable volume [12,15,19]. This method can use the top diameter or the merchantable height as independent variable, combining with *D*, total tree height and stump height [17,18,20]. However, although great progress has been made to estimate the merchantable volume as mentioned above, the majority of the existing models have been developed to estimate tree-level merchantable volume and to date very few studies have been carried out concerning stand-level volume-ratio equations [21].

Although many taper functions have been developed for different tree species, the taper function for Chinese fir is still rare despite a generalized taper function was developed about 20 years ago using a single taper model [22]. However, this taper function cannot estimate the merchantable volume. Recent evidence further indicates that segmented taper models are more accurate and flexible to describe the stem taper [14,15]. To our knowledge, no such taper function was developed for Chinese fir plantation. This study attempts to make up this gap using the taper function developed by Fang et al. [14]. The objectives of this study are to: (i) develop a taper function that can correctly describe the stem taper, estimate stem volume and tree merchantable volume as a baseline in China; (ii) develop a stand-level merchantable volume model to simplify the stand merchantable volume estimation for Chinese fir plantations.

## Materials and Methods

### Ethics Statement

Our study was conducted in Shitai County, China. All the necessary permits for felling trees were obtained through Shitai Forestry Bureau and did not involve endangered or protected species.

### Study area

**Fig 1:**
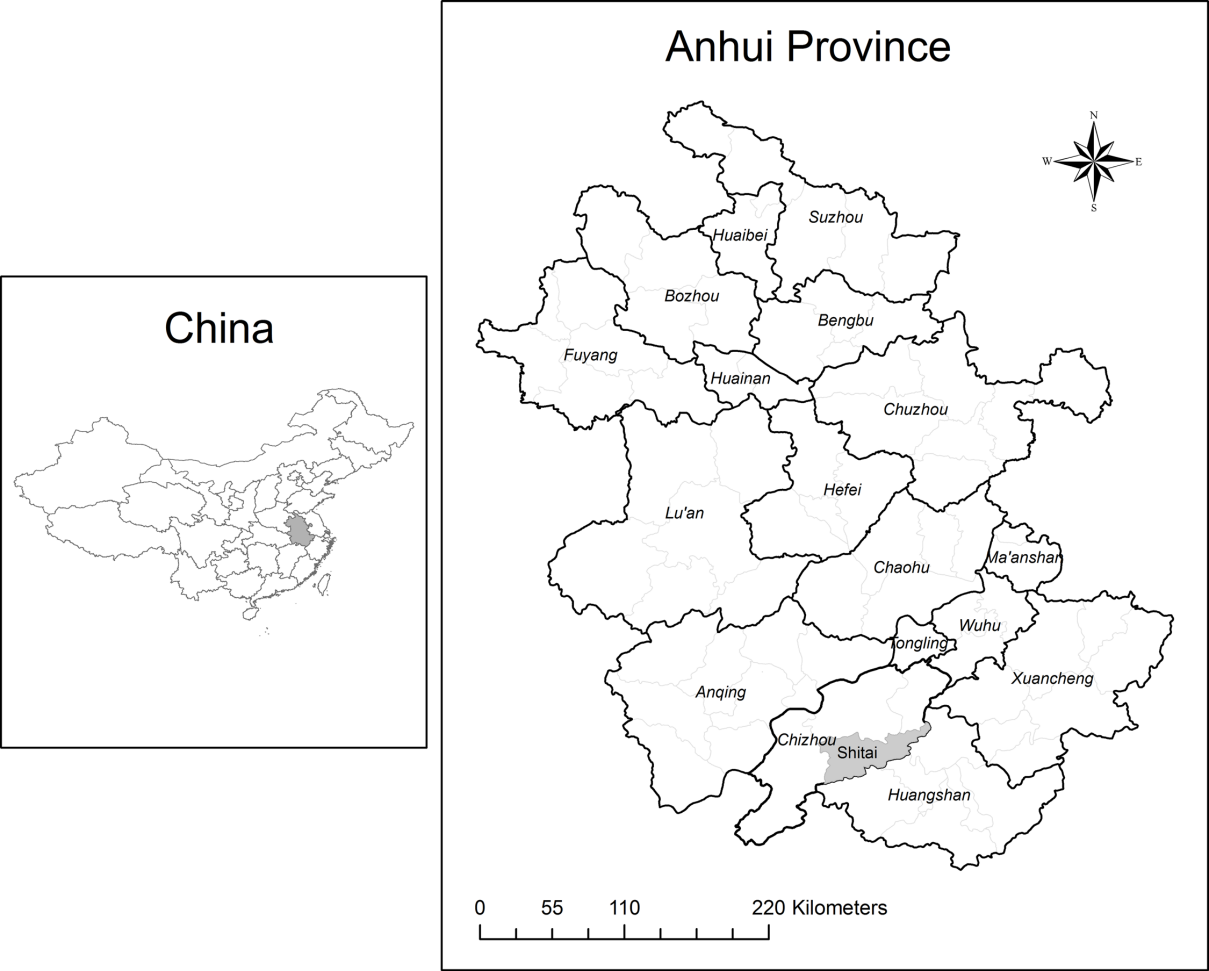
Location of the study area.

The study was conducted in Shitai County (29°59'-30°24' N, 117°12'-117°59' E, Fig 1), Anhui province, China. It is a very mountainous area with an average slope of 66%. It has a forest cover of 80% with an elevation varying between 50 m and 1000 m above the sea level. The region has a mid-subtropical, humid, mountainous climate with seasonal variability [23]. The annual average temperature is 16°C, ranging from 40.9°C in July to -13.2°C in January [24]. The mean annual precipitation is about 1668 mm with high inter-annual variability, and with about 70% of the precipitation occurring during flooding seasons [23]. The average annual sunshine duration is 1704 hours and evaporative capacity is 1256 mm [24].

### Plot design

This study is a part of the Lin^4^carbon project, in which three different types of inventories were established: (1) a land use and forest inventory (LUI/FI) that produces information over the whole extent of the sampling frame based on a systematic grid of 3×3 km; (2) a forest management inventory (FMI) for those stands with forest management information with a 500×500 m systematic grid, and (3) a stand inventory of selected stands (SI) with a 100×100 m systematic grid. Altogether 74 nested circular plots in the Chinese fir plantations were established. Trees with a *D* within 10–20 cm were measured in the 6 m radius circular subplots with a diameter tape to the nearest 0.1 cm. While trees with a *D* > 20 cm were measured in the 10 m radius subplots. In each plot, heights of one or two dominant, one co-dominant and one suppressed trees were measured, respectively. A height-diameter relationship was fitted to the subsample of trees with diameter and height measurements using the model proposed by Pretzsch [25]:

$$H=1.3+{\left(\frac{D}{2.0208+0.3461∙D}\right)}^{3}\;R^{2}{}_{adj}=0.7286,RMSE=1.6006$$ (1)

Where *H* is the total height of the tree in m, *D* is the diameter at breast height (1.3 m aboveground) in cm, *R*^*2*^_adj_ is the coefficient of determination and *RMSE* is the root mean square error.

### Dataset

**Fig 2:**
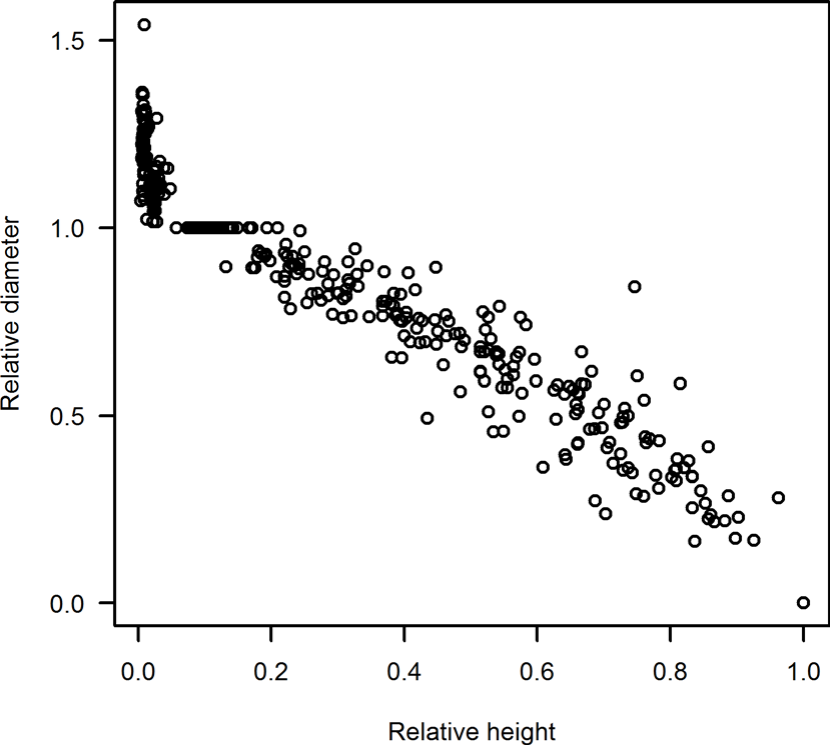
Data points of relative diameter and relative height.

A total of 46 non-forked sample trees were felled throughout the study area, covering the existing range of age, diameter, stand density and site quality. *D* was measured for each tree to the nearest 0.1 cm with a diameter tape before felling. The trees were felled with an average stump height of 0.1 m, and the total tree height was measured to the nearest 0.1 m using a measuring tape. The stem was cut into sections at heights of 0.3 m, 1.3 m, 3 m and 2 m intervals up to a top diameter of 5 cm. Smalian formula was used to calculate the log volumes [8,15]. The treetop was treated as a cone. Total stem volume with bark was obtained by summing the log volumes and treetop volume. The relative diameter against relative height are plotted to detect the anomalies in the data (Fig 2). More summary statistics of the felled trees used for fitting the taper function are given in Table 1.

**Table 1:** Summary statistics of the 46 trees used for developing the taper function.

	*D*(cm)*	*H* (m)*	*V* (m^3^)*
Mean	18.1	12.3	0.1926
Minimum	8.8	6.2	0.0251
Maximum	39.4	22.7	1.0612
S.D.**	6.5	3.2	0.1962

*D is the diameter at breast height (1.3 m aboveground); *H* is the total tree height; *V* is the total tree volume^,^

**S.D. is standard deviation.

### Taper function proposed by Fang et al. [14]

The exponential-segmented-compatible model assumes that the tree stem has three sections with different form factors. The compatible function has three equations: a taper function, a total stem volume equation and a merchantable volume equation. They are expressed:

Taper function:

$$d=c_{1}\sqrt{H^{(k-b_{1})/b_{1}∙{\left(1-q\right)}^{(k-\beta )/\beta }∙\alpha_{1}^{I_{1}+I_{2}}∙\alpha_{2}^{I_{2}}}}$$ (2)

where *I*_1_ = 1, if *p*_1_ ≤ *q* ≤ *p*_2_; 0 otherwise;

*I*_2_ = 1, if *p*_2_ ≤ *q* ≤ 1; 0 otherwise

*p*_1_ and *p*_2_ are the relative height from the ground level where the two inflection points assumed in the model occur.

$$\beta =b_{1}^{1-(I_{1}+I_{2})}∙b_{2}^{I_{1}}∙b_{3}^{I_{2}},\;a_{1}={\left(1-p_{1}\right)}^{\frac{(b_{2}-b_{1})∙k}{b_{1}∙b_{2}}},\;a_{2}={\left(1-p_{2}\right)}^{\frac{(b_{3}-b_{2})∙k}{b_{2}∙b_{3}}}$$

$$r_{0}={\left(1-h_{st}/H\right)}^{\frac{k}{b_{1}}},\;r_{1}={\left(1-p_{1}\right)}^{\frac{k}{b_{1}}},\;r_{2}={\left(1-p_{2}\right)}^{\frac{k}{b_{2}}}$$

$$c_{1}=\sqrt{\frac{a_{0}∙D^{a_{1}}∙H^{a_{2}-k/b_{1}}}{b_{1}∙(r_{0}-r_{1})+b_{2}∙(r_{1}-\alpha_{1}∙r_{2})+b_{3}∙\alpha_{1}∙r_{2}}}$$

Merchantable volume equation:

$$v=c_{1}^{2}∙H^{\frac{k}{b_{1}}}∙[b_{1}∙r_{0}+(I_{1}+I_{2})∙(b_{2}-b_{1})∙r_{1}+I_{2}∙(b_{3}-b_{2})∙\alpha_{1}∙r_{2}-\beta ∙{\left(1-q\right)}^{k/\beta }∙\alpha_{1}^{I_{1}+I_{2}}∙\alpha_{2}^{I_{2}}]$$ (3)

Total volume equation:

$$V=a_{0}∙D^{a_{1}}∙H^{a_{2}}$$ (4)

Where *d* = top diameter with bark (cm) at height *h*;

*D* is the diameter at breast height (1.3 m aboveground, cm);

*H* = total tree height (m);

*h* = height aboveground (m) to top diameter *d*;

*h*_*st*_ = stump height (m);

*v* = merchantable volume with bark from the stump to the height *h* (m^3^);

*V* = total tree volume (m^3^);

*k* = π/40000, a metric constant for converting from the diameter squared in cm^2^ to cross-section area in m^2^;

*q* = *h/H*.

### Modelling

The compatible system of Fang et al. [14] has two components: a taper function and a total volume equation. The system is composed by the endogenous variables (variables included on the left-hand side of the equations) *d* and *v*, which are assumed to be determined by the model structure. The remaining variables–*D*, *H*, *h*, and *h*_*st*_−are exogenous, i.e., they are independent variables. An endogenous variable in one equation of the system can also appear on the right-hand side of the other equation.

The number of observations in each equation are not equal because there is more than one diameter observation for each tree but only one observation for total stem volume. However, the simultaneous fitting of both equations requires the number of observations of the two endogenous variables (*d* and *v*) to be equal. To solve this problem we created a special structure for the data: the total volume of the tree was assigned to each diameter observation on the same tree. The inverse of the number of observations in each tree (*n*_*i*_) was then used as weight of the total volume in the fitting process.

Estimation of the parameters was carried out with the MODEL procedure of SAS/ETS^®^ [26], in which several methods of parameter estimation are available. We used the *nonlinear seemingly unrelated regression* (NSUR) technique, in which the random errors of the equations are correlated but the equations are not really simultaneous (none of the endogenous variables in one equation of the system appears as dependent on the left-hand side of the other equation).

### Stand-level volume model construction

To simplify the stand-level volume estimation, the volume ratio approach was used. This approach was firstly introduced by Burkhart [27] and its use requires fitting a total stand volume equation (*V*_*s*_) and a stand volume ratio equation (*R*_*i*_). As Gregoire and Schabenberger [28] pointed out, both terms (*R*_*i*_ and *V*_*s*_) should be included in the same expression, in which case the total volume becomes a special case of the volume ratio equation when the top diameter limit is equal to zero. Therefore, a composite model including stand total volume and a volume ratio equation to estimate merchantable stand volume to a top diameter limit was fitted.

The commonly used allometric model, which includes stand basal area (*G*, m^2^ha^-1^) and dominant height (*H*_*d*_, m, the average height of the 100 largest trees per hectare) as independent variables, was selected to determine the total stand volume. A modification of the exponential ratio model originally developed by Van DeusenSullivan and Matvey [29] was selected as volume ratio equation. This model was developed for individual trees; therefore, the original tree variables were replaced by equivalent stand variables (tree height was replaced by dominant height and diameter at breast height by quadratic mean diameter).

$$V_{S}=c_{0}∙G^{c_{1}}∙H_{d}^{c_{2}}∙exp(c_{3}∙d_{i}^{c_{4}}{∙d}_{g}^{c_{5}})$$ (5)

Where *V*_*s*_ is the merchantable stand volume (m^3^ha^-1^) to a top diameter *d*_*i*_; *G* is the stand basal area (m^2^ha^-1^); *H*_*d*_ is the dominant height (m); *d*_*g*_ is the quadratic mean diameter (cm) and *c*_*i*_ are parameters to be fitted.

Total and merchantable stand volumes were calculated by aggregating tree volumes (upper limit diameters ranging between 0 and 30 cm) calculated by using the fitted model proposed by Fang et al. [14] (Eqs 3 and 4) for each tree in each one of the 74 nested circular plots.

### Multicollinearity, autocorrelation and heteroscedasticity

There are several problems associated with stem taper and volume equation analysis that violate the fundamental least squares assumption of independence and equal distribution of errors with zero mean and constant variance: multicollinearity, autocorrelation and heteroscedasticity are three of the most important. Although the least squares estimates of regression coefficients remain unbiased and consistent under the presence of multicollinearity, autocorrelation and heteroscedasticity, they are no longer efficient. These problems may seriously affect the standard errors of the coefficients, invalidating statistical tests using *t* or *F* distributions and confidence intervals. Thus, appropriate statistical procedures should be used in model fitting to avoid the problems of heteroscedasticity and autocorrelated errors, and models with low multicollinearity should be selected whenever possible.

Multicollinearity refers to the existence of high intercorrelations among the independent variables in multiple linear or nonlinear regression analysis, because some of the variables represent or measure similar phenomena. One of the main sources of multicollinearity is the use of overcomplicated models that include several polynomial and cross-product terms. However, the model of Fang et al. [14] does not include these terms, so multicollinearity is not a real problem.

Since the database contains multiple observations for each tree (i.e., hierarchical data), one may expect autocorrelation within the residuals of each individual, which violates the assumption of independent error terms. We have corrected the autocorrelation using a modified continuous autoregressive error structure (mCAR(m)), which accounted for the distance between measurements and the relative position on the stem they were taken from. To account for autocorrelation, a general m-order mCAR(m) model form, which expands the error terms in the following way, was used:

$$e_{ij}={\sum_{k=1}^{m}d}_{k}\rho_{k}^{h_{ij}-h_{ij-k}}e_{ij-k}+\varepsilon_{ij}$$ (6)

where *e*_*ij*_ is the *j*th ordinary residual on the *i*th individual (i.e., the difference between the observed and the estimated diameters of the tree *i* at height measurement *j*), *d*_k_ = 1 for *j* > *k* and it is zero for *j* **≤** *k*, *ρ*_*k*_ is the *k*-order continuous autoregressive parameter to be estimated, *h*_*ij*_-*h*_*ij*-*k*_ is the distance separating the *j*th from the *j*th-*k* observations within each tree, *h*_*ij*_ > *h*_*ij*-*k*_and *ε*_*ij*_ are independent normal random variables with a mean value of zero. In this study, we used a second order continuous autoregressive correction.

To test for the presence of autocorrelation we have used the Durbin-Watson test. The modified mCAR(m) error structure was programmed in the MODEL procedure of SAS/ETS^®^ [26], which allows for dynamic updating of the residuals.

Forest modellers are often faced with the problem of heteroscedasticity in their data, which would lead to non-minimum variance parameter estimates and unreliable predictor intervals. This is especially true in the construction of volume equations. The solution to the problem is to weight each observation during the fitting process by the inverse of its variance ($\sigma_{i}^{2}$). If the variance is unknown, the problem becomes one of estimating the proper weight for each observation. Although several assumptions about the nature of the heteroscedasticity problem in the construction of volume equations that depend on two variables were suggested, it is often assumed that the variance of the error of the *i*th individual can be modelled as a power function of $D_{i}^{2}H_{i}$ for individual tree volumes (e.g. [30,31]), i.e., $\sigma_{i}^{2}={\left(D_{i}^{2}H_{i}\right)}^{k_{1}}$ and *GH*_*d*_ for stand volumes, i.e., $\sigma_{i}^{2}={\left(G_{i}H_{d}{}_{i}\right)}^{k_{2}}$. The most reasonable values of the exponential terms *k*_*j*_ should provide the most homogeneous studentized residual plot. It can be obtained by iteratively testing different values of *k* (e.g., from 0.1 to 2), or optimizing the value using the method suggested by Harvey [32], which consists of using the estimated errors of the unweighted model (${\hat{e}}_{i}$) as the dependent variable in the error variance model.

The *k*_*j*_ parameters were estimated using linear regression. The weighting factor for heteroscedasticity $1/{\left(X_{i}\right)}^{k_{j}}$, along with the correction for the special structure of the data that we used, were multiplied and programmed in the MODEL procedure of SAS/ETS^®^ by specifying $resid.V=resid.V/\sqrt{n_{i}{\left(X_{i}\right)}^{k_{j}}}$, where *n*_*i*_ is the number of observations in each tree (note that the residual –*resid*.*V*– is multiplied by $1/\sqrt{n_{i}{\left(X_{i}\right)}^{k_{j}}}$ because the latter is acting on the residual before it is squared).

## Results and Discussion

### Taper function

**Fig 3:**
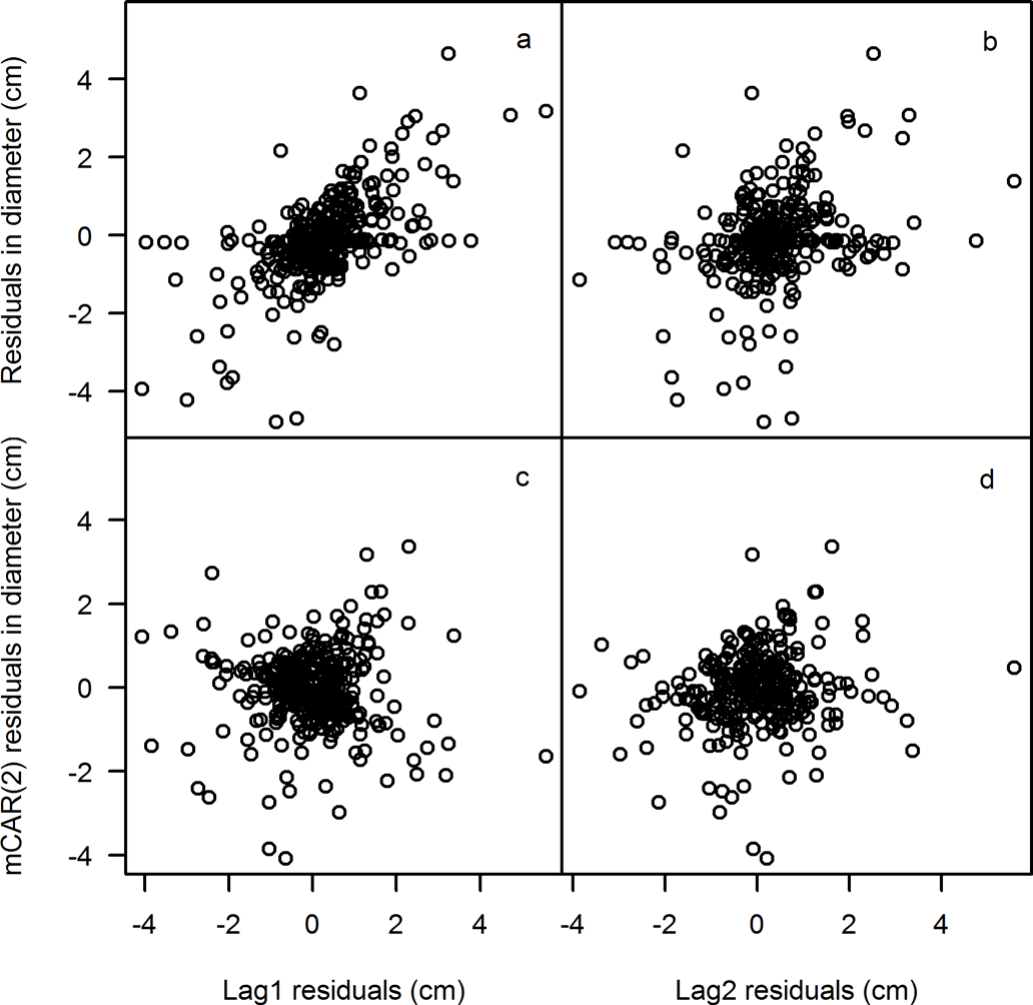
Residuals in diameter plotted against lag1 and lag2 without autocorrelation (a, b), and against lag 1 and lag2 with continuous time autoregressive error structure of second order (mCAR(2), c, d).

First, the taper function was fitted without autocorrelation. Strong linear relationships between the residuals in diameter and lag 1-residuals and lag 2-residuals were found (Fig 3A and 3B). This indicates that there is a strong autocorrelation among the multiple observations from each tree. Therefore, a second-order continuous autoregressive error structure (mCAR(2)) was conducted to correct the autocorrelation. After the correction, the linear trend between the residuals in diameter and lag 1-residuals and lag 2-residucals disappeared (Fig 3C and 3D). Although the fitted curve shapes were not significantly different from the model without autocorrelation, the autocorrelation correction could improve the interpretation of the statistical properties of the model [15].

The coefficient estimates after autocorrelation and their approximated standard errors are given in Table 2, and the goodness-of-fit statistics of the taper function and the total stem volume are shown in Table 3. All the coefficients were significant at a 0.001 level. The fitted system proposed by Fang et al. [14] showed very good fitness of the data, and could explain more than 98% of the total variations of *d* and *V*.

**Table 2:** Coefficient estimates for the taper functions.

Coefficients	Estimates	Standard error	*p*
*a*_0_	7.1×10^−5^	3.5×10^−6^	<0.001
*a*_1_	1.7512	0.0288	<0.001
*a*_2_	1.0417	0.0402	<0.001
*b*_1_	4.2×10^−6^	3.4×10^−7^	<0.001
*b*_2_	3.1×10^−5^	5.3×10^−7^	<0.001
*b*_3_	3.2×10^−5^	1.3×10^−6^	<0.001
*p*_1_	0.0202	0.0012	<0.001
*p*_2_	0.6577	0.1548	<0.001
*ρ*_1_	0.7483	0.0443	<0.001
*ρ*_2_	0.3900	0.0680	<0.001

**Table 3:** Goodness-of-fit statistics of the taper function and volume model.

	*RMSE*	*R*^*2*^_adj_	Weighting factor
Eq (2)	1.0481	0.9856	---
Eq (4)	0.0243	0.9878	1/(*D*^*2*^_*i*_*H*_*i*_)^1.1987^

RMSE is root mean square error and *R*^*2*^_adj_ is adjusted determination coefficient.

**Fig 4:**
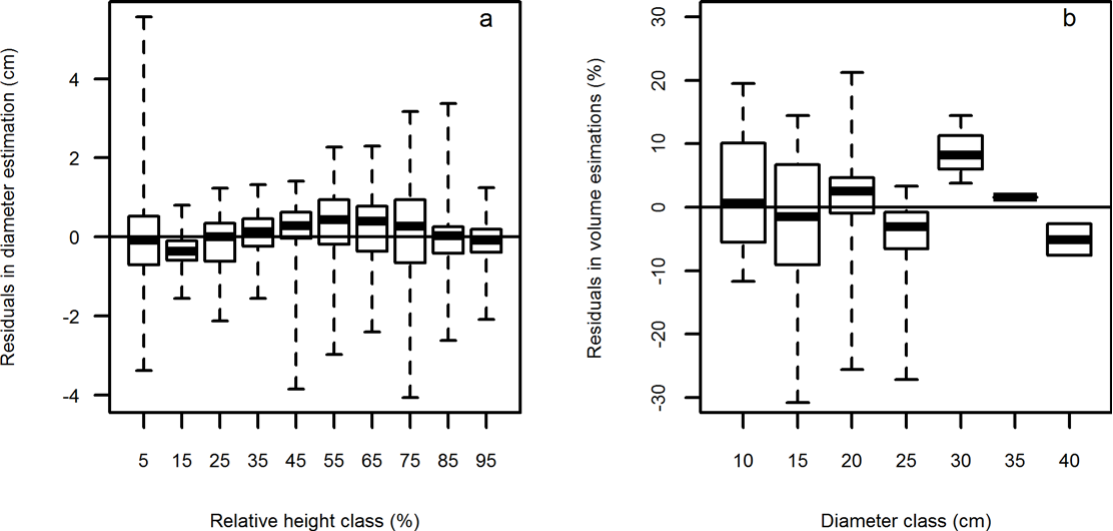
Boxplot of: (a) residuals in diameter against relative height classes and (b) residuals in volume estimation against diameter classes. The horizontal line represents 0 in residuals.

Although the goodness-of-fit could reflect the behavior of the data evaluated, they may not indicate the best practical uses because the larger residuals compensated the smaller residuals [15]. Therefore, the goodness should be determined based on the analysis of the model’s behavior for different stem sections. For this purpose, the residuals of diameter were plotted against the relative height classes with an interval of 10%, and the residuals of volume were plotted against the diameter classes with an interval of 5 cm (Fig 4). The residuals in diameter ranged from -4.1 cm to 5.6 cm with an average of 0.02 cm. This demonstrates that on average the Fang et al. [14] function performed well in the prediction of bole diameter. As expected, the prediction of diameter for lower section closest to the ground was less precise than that of upper sections. This has also been observed in other tree species using different taper functions [15,33]. This is mainly due to the irregular shape of the stem section close to the ground level. The taper function had the most accurate prediction in diameter for relative height classes between 15% and 35% because of regular stem shape between these heights. For relative height classes higher than 35%, the precision decreased with the increase of the relative height.

The taper function also performed well in total tree volume prediction, with the average residuals ranging from -6% to 8% for different diameter classes (Fig 4B). However, we do not recommend the application of our model for trees with diameter larger than 30 cm due to the small sample size for trees of these dimensional classes. Moreover, the Chinese fir plantations in our study area are commonly harvested at the age of 20–30 years old, when only few trees larger than 30 cm can be found. Therefore, our model is dimensionally valid for most of the dimensional classes during the rotation period in the study area.

### Stand-level merchantable volume model

The composite Eq (5) was fitted to estimate stand-level merchantable volume. All the coefficients were significant at 0.001 level (Table 4). The model explained more than 99% of the variance of stand volume with a RMSE of 4.3 m^3^ha^-1^. When *d*_*i*_ equals to zero, the stand merchantable volume equals to the total stand volume. The results highlighted the efficiency of total stand-level volume estimation by using the stand-level model developed here, as *G* can efficiently be estimated by using angle-count techniques (i.e., with a dendrometer) and, similarly, the dominant height can be more efficiently acquired than individual tree heights. For example, the average measuring time of forest inventory among the 74 plots was about 33 minutes, not including tree harvest in some plots. However, it took just 7 minutes to measure stand basal area using dendrometer and dominant heights.

**Table 4:** Coefficient estimates of stand-level volume model Eq (5).

Coefficients	Estimates	Approx. S.E.	*p*	*R*^*2*^_adj_	*RMSE*	Weighting factor
*c*_0_	1.2936	0.0361	<0.001			
*c*_1_	0.9808	0.0029	<0.001			
*c*_2_	0.5846	0.0119	<0.001	0.9953	4.2997	1/(*G*_*i*_*H*_*di*_)^0.7761^
*c*_3_	-0.7332	0.0736	<0.001			
*c*_4_	3.8938	0.0308	<0.001			
*c*_5_	-3.8075	0.0466	<0.001			

S.E. is standard error and *RMSE* is root mean square error. *p* is the approximate probability value and *R*^*2*^_adj_ is adjusted determination coefficient.

**Fig 5:**
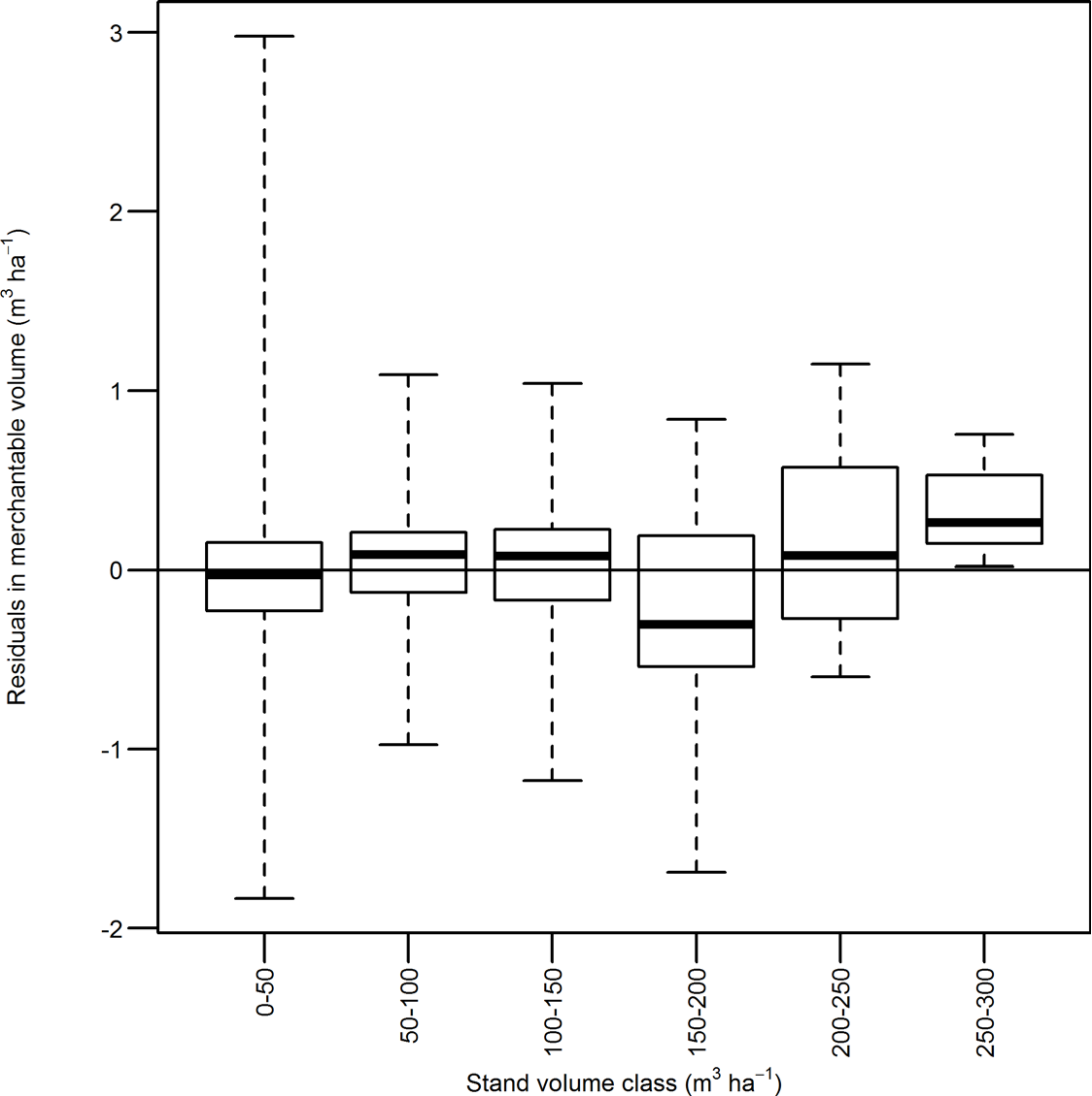
Boxplot of residuals of Eq (5) in stand merchantable volume estimation against stand volume classes.

Boxplot of residuals against the volume classes were drawn in Fig 5. The residuals of different volume classes ranged from -1.8 m^3^ha^-1^ to 2.9 m^3^ha^-1^ with a mean value of 0.03 m^3^ha^-1^ (Fig 4). Eq (5) had the most accurate prediction for volume class 50–100 m^3^ha^-1^, while it had the least accurate prediction for volume class 0–50 m^3^ha^-1^. Eq (5) overestimated stand merchantable volume for volume class 150–200 m^3^ha^-1^, but slightly underestimated merchantable volume for other volume classes.

### Model performance comparisons in the estimation of stand level merchantable volume

**Fig 6:**
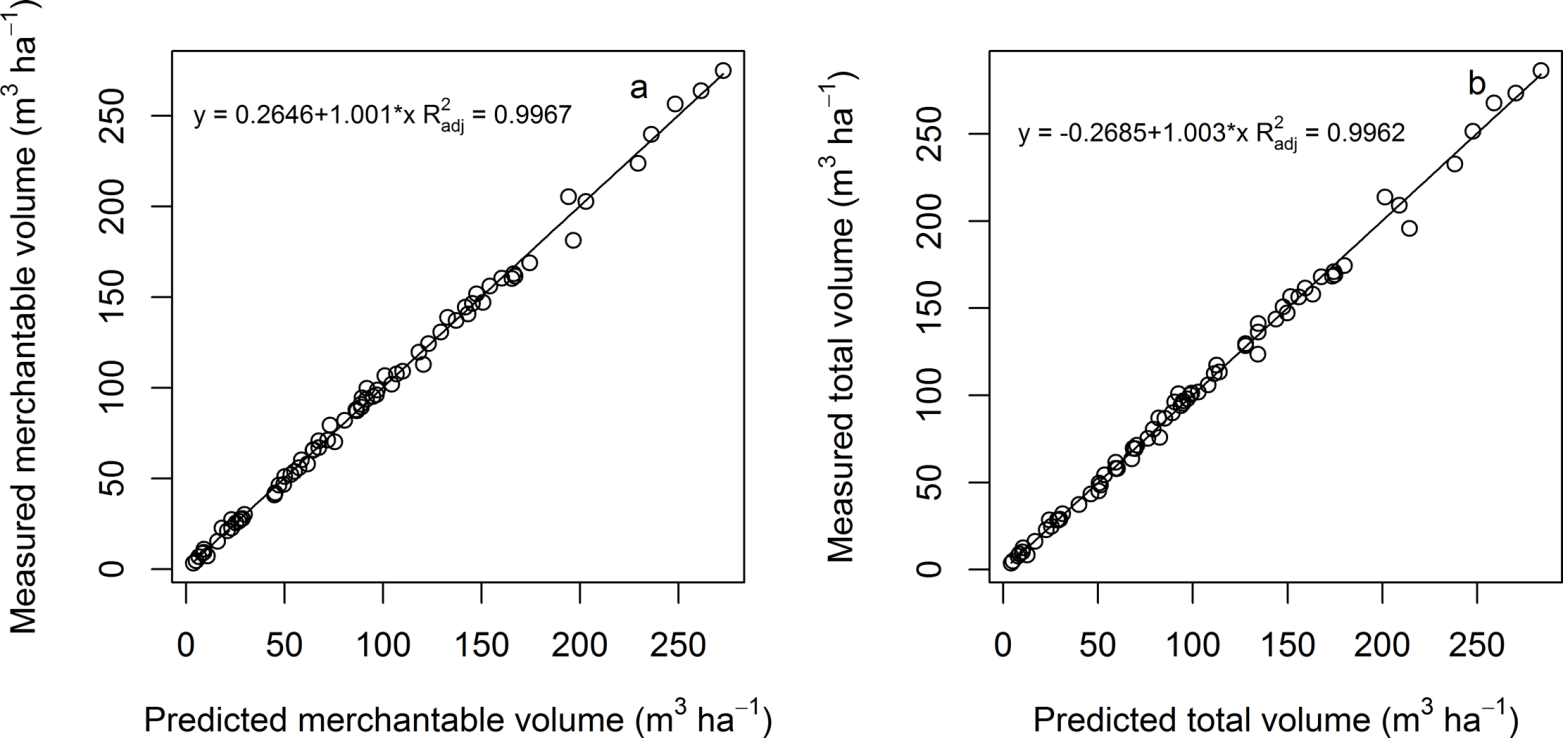
Relationship between (a) measured total stand volume and predicted total stand volume, (b) measured stand merchantable volume and predicted stand merchantable volume for local top diameter of 7 cm.

Two practical examples are presented to test the performance of the stand level model in predicting the stand merchantable volume. The first example defines the top diameter as zero, in which case, the merchantable volume equals total stand volume, which was presented in the *x*-axis (Fig 6A). The measured stand volume calculated using Eq (4) for each individual tree considering the expansion factors was presented in *y*-axis. High correlation was found between the two estimates (*R*^*2*^_adj_ > 0.99). The second example was to predict the merchantable volume according to the local top diameter of 7 cm. Similarly, there was a strong relationship between the measured merchantable and predicted merchantable volume (*R*^*2*^_adj_ > 0.99, Fig 6B). These results highly demonstrated the wide application of Eq (5) in predicting stand merchantable volume for different top diameters. The results also highlighted the simplification of using Eq (5) to total stand volume by setting top diameter to zero. The calculated ratio of merchantable stand volume and total stand volume was 94.5% (range from 88.0% to 99.0%) based on the 74 plots if the top diameter was set to 7 cm.

### Construction of stand-level volume table

To make our models more applicable, a stand-level volume table was constructed for Chinese fir plantation for local study area using stand basal area and dominant height based on the Eq (5). As mentioned above, stand basal area could be easily measured using a dendrometer without moving up and down in the stand, and just measure few heights of dominant trees based different plot sizes. Afterwards, local forest manager could “read” the stand volume directly from Table 5. Stand merchantable volume could be also estimated by multiplying the ratio of stand merchantable volume and total stand volume (94.5%).

**Table 5:** Stand-level volume table for Chinese fir plantations.

*H*_*d*_ (m) G (m^2^ ha^-1^)	8	9	10	11	12	13	14	15	16	17	18	19	20	21	22
6	25.3	27.1	28.8	30.5	32.1	33.6	35.1	36.5	37.9	39.3	40.6	41.9	43.2	44.5	45.7
7	29.4	31.5	33.5	35.4	37.3	39.1	40.8	42.5	44.1	45.7	47.3	48.8	50.3	51.7	53.2
8	33.5	35.9	38.2	40.4	42.5	44.6	46.5	48.4	50.3	52.1	53.9	55.6	57.3	59	60.6
9	37.6	40.3	42.9	45.4	47.7	50	52.2	54.4	56.5	58.5	60.5	62.4	64.3	66.2	68
10	41.7	44.7	47.6	50.3	52.9	55.5	57.9	60.3	62.6	64.9	67.1	69.2	71.3	73.4	75.4
11	45.8	49.1	52.2	55.2	58.1	60.9	63.6	66.2	68.7	71.2	73.6	76	78.3	80.6	82.8
12	49.9	53.5	56.9	60.1	63.3	66.3	69.2	72.1	74.9	77.6	80.2	82.8	85.3	87.8	90.2
13	54	57.8	61.5	65	68.4	71.7	74.9	78	81	83.9	86.8	89.5	92.3	94.9	97.6
14	58.1	62.2	66.2	70	73.6	77.1	80.5	83.9	87.1	90.2	93.3	96.3	99.2	102.1	104.9
15	62.1	66.6	70.8	74.9	78.8	82.5	86.2	89.7	93.2	96.5	99.8	103	106.2	109.2	112.3
16	66.2	70.9	75.4	79.7	83.9	87.9	91.8	95.6	99.3	102.9	106.4	109.8	113.1	116.4	119.6
17	70.3	75.3	80	84.6	89	93.3	97.4	101.5	105.4	109.2	112.9	116.5	120	123.5	126.9
18	74.3	79.6	84.7	89.5	94.2	98.7	103.1	107.3	111.4	115.5	119.4	123.2	127	130.6	134.2
19	78.4	83.9	89.3	94.4	99.3	104.1	108.7	113.1	117.5	121.7	125.9	129.9	133.9	137.7	141.5
20	82.4	88.3	93.9	99.3	104.4	109.4	114.3	119	123.6	128	132.4	136.6	140.8	144.9	148.8
21	86.4	92.6	98.5	104.1	109.6	114.8	119.9	124.8	129.6	134.3	138.9	143.3	147.7	152	156.1
22	90.5	96.9	103.1	109	114.7	120.2	125.5	130.6	135.7	140.6	145.3	150	154.6	159	163.4
23	94.5	101.2	107.7	113.8	119.8	125.5	131.1	136.5	141.7	146.8	151.8	156.7	161.5	166.1	170.7
24	98.5	105.6	112.3	118.7	124.9	130.9	136.7	142.3	147.8	153.1	158.3	163.4	168.3	173.2	178
25	102.6	109.9	116.8	123.5	130	136.2	142.2	148.1	153.8	159.3	164.8	170	175.2	180.3	185.3
26	106.6	114.2	121.4	128.4	135.1	141.6	147.8	153.9	159.8	165.6	171.2	176.7	182.1	187.4	192.5
27	110.6	118.5	126	133.2	140.2	146.9	153.4	159.7	165.9	171.8	177.7	183.4	189	194.4	199.8
28	114.6	122.8	130.6	138.1	145.3	152.2	159	165.5	171.9	178.1	184.1	190	195.8	201.5	207
29	118.6	127.1	135.2	142.9	150.4	157.6	164.5	171.3	177.9	184.3	190.6	196.7	202.7	208.5	214.3
30	122.6	131.4	139.7	147.7	155.4	162.9	170.1	177.1	183.9	190.5	197	203.3	209.5	215.6	221.5
31	126.6	135.7	144.3	152.6	160.5	168.2	175.7	182.9	189.9	196.8	203.5	210	216.4	222.6	228.8
32	130.6	140	148.9	157.4	165.6	173.5	181.2	188.7	195.9	203	209.9	216.6	223.2	229.7	236
33	134.6	144.2	153.4	162.2	170.7	178.8	186.8	194.5	201.9	209.2	216.3	223.3	230.1	236.7	243.3
34	138.7	148.5	158	167	175.7	184.2	192.3	200.2	207.9	215.4	222.8	229.9	236.9	243.8	250.5
35	142.6	152.8	162.5	171.8	180.8	189.5	197.9	206	213.9	221.6	229.2	236.5	243.7	250.8	257.7
36	146.6	157.1	167.1	176.7	185.9	194.8	203.4	211.8	219.9	227.9	235.6	243.2	250.6	257.8	264.9
37	150.6	161.4	171.6	181.5	190.9	200.1	208.9	217.5	225.9	234.1	242	249.8	257.4	264.8	272.1
38	154.6	165.7	176.2	186.3	196	205.4	214.5	223.3	231.9	240.3	248.4	256.4	264.2	271.9	279.4
39	158.6	169.9	180.7	191.1	201.1	210.7	220	229.1	237.9	246.5	254.8	263	271	278.9	286.6
40	162.6	174.2	185.3	195.9	206.1	216	225.5	234.8	243.9	252.7	261.2	269.6	277.8	285.9	293.8

*G* is stand basal area (m^2^ ha^-1^), *H*_*d*_ is the mean dominant height of the 100 largest trees per hectare (m).

## Conclusions

The compatible taper function proposed by Fang et al. [14] was analysed in this study and it fitted well to our data. A modified second-order continuous autoregressive error structure corrected the inherent serial autocorrelation of different observations in one tree. The diameter and volume were fitted simultaneously after autocorrelation correction. Fang et al. [14] performed well in diameter and total stem volume prediction. A modified stand-level merchantable volume (Eq 5) based on the volume ratio approach was developed including basal area, dominant height, quadratic mean diameter and top diameter as regressors. One quarter of the measuring time of total trees in each inventory plot was used when measuring basal area and dominant height for total stand volume, demonstrating the efficiency of using the stand-level volume model to estimate stand merchantable volume and total stand volume. A stand volume table was also proposed for local forest managers to “read” stand volume. This could be a useful tool for local forest managers to increase their work efficiency and simplify the fieldwork.

## Supporting Information

S1 Table Diameter at different heights of the 46 felled Chinese fir trees. (PDF)

S2 Table Stand characteristics of the 74 inventory plots. (PDF)

S1 Text SAS code for the compatible taper function and stand-level merchantable volume modelling. (PDF)
