# Supplementary material for: Development of a Compatible Taper Function and Stand-Level Merchantable Volume Model for Chinese Fir Plantations
Source: PLoS One. 2016 Jan 22;11(1):e0147610. doi: 10.1371/journal.pone.0147610 (PMC4723312; doi:10.1371/journal.pone.0147610)
Supplement: S1 Table — (PDF) [file pone.0147610.s001.pdf]

tree	hi	di	v	D	H	h0
1	0.1	22.2	0.138097462	18.3	10.2	0.1
1	0.3	20.4	0.138097462	18.3	10.2	0.1
1	1.3	18.3	0.138097462	18.3	10.2	0.1
1	3	16	0.138097462	18.3	10.2	0.1
1	5	12.8	0.138097462	18.3	10.2	0.1
1	7	8.5	0.138097462	18.3	10.2	0.1
1	9	4	0.138097462	18.3	10.2	0.1
1	10.2	0.001	0.138097462	18.3	10.2	0.1
2	0.1	16.5	0.054330782	13.1	7.8	0.1
2	0.3	15.2	0.054330782	13.1	7.8	0.1
2	1.3	13.1	0.054330782	13.1	7.8	0.1
2	3	10.8	0.054330782	13.1	7.8	0.1
2	5	7.3	0.054330782	13.1	7.8	0.1
2	7.8	0.001	0.054330782	13.1	7.8	0.1
3	0.1	12.2	0.025086756	9.6	6.2	0.1
3	0.3	10.6	0.025086756	9.6	6.2	0.1
3	1.3	9.6	0.025086756	9.6	6.2	0.1
3	3	6.9	0.025086756	9.6	6.2	0.1
3	5	3.4	0.025086756	9.6	6.2	0.1
3	6.2	0.001	0.025086756	9.6	6.2	0.1
4	0.1	25	0.191793171	19.8	12.7	0.1
4	0.3	22.3	0.191793171	19.8	12.7	0.1
4	1.3	19.8	0.191793171	19.8	12.7	0.1
4	3	17.8	0.191793171	19.8	12.7	0.1
4	5	14.9	0.191793171	19.8	12.7	0.1
4	7	12.3	0.191793171	19.8	12.7	0.1
4	9	8.5	0.191793171	19.8	12.7	0.1
4	11	4.3	0.191793171	19.8	12.7	0.1
4	12.7	0.001	0.191793171	19.8	12.7	0.1
5	0.1	17.1	0.077908356	14.4	9.5	0.1
5	0.3	16.1	0.077908356	14.4	9.5	0.1
5	1.3	14.4	0.077908356	14.4	9.5	0.1
5	3	12.4	0.077908356	14.4	9.5	0.1
5	5	9.7	0.077908356	14.4	9.5	0.1
5	7	5.2	0.077908356	14.4	9.5	0.1
5	9.5	0.001	0.077908356	14.4	9.5	0.1
6	0.1	15	0.045480562	12	9.1	0.1
6	0.3	13.4	0.045480562	12	9.1	0.1
6	1.3	12	0.045480562	12	9.1	0.1
6	3	10.5	0.045480562	12	9.1	0.1
6	5	5.5	0.045480562	12	9.1	0.1
6	9.1	0.001	0.045480562	12	9.1	0.1

7	0.1	26.6	0.280910102	22.5	15.6	0.1
7	0.3	24.3	0.280910102	22.5	15.6	0.1
7	1.3	22.5	0.280910102	22.5	15.6	0.1
7	3	20.9	0.280910102	22.5	15.6	0.1
7	5	17.2	0.280910102	22.5	15.6	0.1
7	7	15.5	0.280910102	22.5	15.6	0.1
7	9	12.6	0.280910102	22.5	15.6	0.1
7	11	9.3	0.280910102	22.5	15.6	0.1
7	13	5.7	0.280910102	22.5	15.6	0.1
7	15.6	0.001	0.280910102	22.5	15.6	0.1
8	0.1	19.2	0.119799138	16.3	10.8	0.1
8	0.3	17.9	0.119799138	16.3	10.8	0.1
8	1.3	16.3	0.119799138	16.3	10.8	0.1
8	3	14.4	0.119799138	16.3	10.8	0.1
8	5	12.5	0.119799138	16.3	10.8	0.1
8	7	9.4	0.119799138	16.3	10.8	0.1
8	9	5.5	0.119799138	16.3	10.8	0.1
8	10.8	0.001	0.119799138	16.3	10.8	0.1
9	0.1	18.4	0.120246501	14.8	13.6	0.1
9	0.3	16.6	0.120246501	14.8	13.6	0.1
9	1.3	14.8	0.120246501	14.8	13.6	0.1
9	3	13.8	0.120246501	14.8	13.6	0.1
9	5	11.9	0.120246501	14.8	13.6	0.1
9	7	10.1	0.120246501	14.8	13.6	0.1
9	9	8.3	0.120246501	14.8	13.6	0.1
9	11	5.3	0.120246501	14.8	13.6	0.1
9	13.6	0.001	0.120246501	14.8	13.6	0.1
10	0.1	16.1	0.072125312	14	11.8	0.1
10	0.3	15.5	0.072125312	14	11.8	0.1
10	1.3	14	0.072125312	14	11.8	0.1
10	3	11.2	0.072125312	14	11.8	0.1
10	5	9.7	0.072125312	14	11.8	0.1
10	9	6.2	0.072125312	14	11.8	0.1
10	11.8	0.001	0.072125312	14	11.8	0.1
11	0.1	21.9	0.189799556	19.6	13.6	0.1
11	0.3	19.9	0.189799556	19.6	13.6	0.1
11	1.3	19.6	0.189799556	19.6	13.6	0.1
11	3	16.8	0.189799556	19.6	13.6	0.1
11	5	15	0.189799556	19.6	13.6	0.1
11	7	13.1	0.189799556	19.6	13.6	0.1
11	9	10.1	0.189799556	19.6	13.6	0.1
11	11	6.4	0.189799556	19.6	13.6	0.1
11	13.6	0.001	0.189799556	19.6	13.6	0.1

12	0.1	18.6	0.09464366	14.8	11.1	0.1
12	0.3	16.3	0.09464366	14.8	11.1	0.1
12	1.3	14.8	0.09464366	14.8	11.1	0.1
12	3	12.2	0.09464366	14.8	11.1	0.1
12	5	10.7	0.09464366	14.8	11.1	0.1
12	7	8.6	0.09464366	14.8	11.1	0.1
12	9	5.7	0.09464366	14.8	11.1	0.1
12	11.1	0.001	0.09464366	14.8	11.1	0.1
13	0.1	18.1	0.083964287	14.2	9.5	0.1
13	0.3	15.5	0.083964287	14.2	9.5	0.1
13	1.3	14.2	0.083964287	14.2	9.5	0.1
13	3	12.9	0.083964287	14.2	9.5	0.1
13	5	10.8	0.083964287	14.2	9.5	0.1
13	7	7.1	0.083964287	14.2	9.5	0.1
13	9.5	0.001	0.083964287	14.2	9.5	0.1
14	0.1	18.5	0.067960032	12	10.5	0.1
14	0.3	15.5	0.067960032	12	10.5	0.1
14	1.3	12	0.067960032	12	10.5	0.1
14	3	10.2	0.067960032	12	10.5	0.1
14	5	8.6	0.067960032	12	10.5	0.1
14	7	7	0.067960032	12	10.5	0.1
14	9	5	0.067960032	12	10.5	0.1
14	10.5	0.001	0.067960032	12	10.5	0.1
15	0.1	16.4	0.084672166	13.7	11.7	0.1
15	0.3	14.6	0.084672166	13.7	11.7	0.1
15	1.3	13.7	0.084672166	13.7	11.7	0.1
15	3	12	0.084672166	13.7	11.7	0.1
15	5	10.3	0.084672166	13.7	11.7	0.1
15	7	8.1	0.084672166	13.7	11.7	0.1
15	9	6	0.084672166	13.7	11.7	0.1
15	11.7	0.001	0.084672166	13.7	11.7	0.1
16	0.1	31.3	0.411484549	26.3	16.2	0.1
16	0.3	28.9	0.411484549	26.3	16.2	0.1
16	1.3	26.3	0.411484549	26.3	16.2	0.1
16	3	24.5	0.411484549	26.3	16.2	0.1
16	5	21.3	0.411484549	26.3	16.2	0.1
16	7	18.3	0.411484549	26.3	16.2	0.1
16	9	15.1	0.411484549	26.3	16.2	0.1
16	11	12.2	0.411484549	26.3	16.2	0.1
16	13	8.8	0.411484549	26.3	16.2	0.1
16	15	4.4	0.411484549	26.3	16.2	0.1
16	16.2	0.001	0.411484549	26.3	16.2	0.1
17	0.1	24.1	0.203051697	17.8	16.6	0.1

17	0.3	19.5	0.203051697	17.8	16.6	0.1
17	1.3	17.8	0.203051697	17.8	16.6	0.1
17	3	16.7	0.203051697	17.8	16.6	0.1
17	5	14.7	0.203051697	17.8	16.6	0.1
17	7	13.5	0.203051697	17.8	16.6	0.1
17	9	11.3	0.203051697	17.8	16.6	0.1
17	11	9.9	0.203051697	17.8	16.6	0.1
17	13	7.7	0.203051697	17.8	16.6	0.1
17	16.6	0.001	0.203051697	17.8	16.6	0.1
18	0.1	25.9	0.299604581	23.6	14.4	0.1
18	0.3	25.6	0.299604581	23.6	14.4	0.1
18	1.3	23.6	0.299604581	23.6	14.4	0.1
18	3	20.5	0.299604581	23.6	14.4	0.1
18	5	18	0.299604581	23.6	14.4	0.1
18	7	16.1	0.299604581	23.6	14.4	0.1
18	9	13.4	0.299604581	23.6	14.4	0.1
18	11	10.1	0.299604581	23.6	14.4	0.1
18	13	5.4	0.299604581	23.6	14.4	0.1
18	14.4	0.001	0.299604581	23.6	14.4	0.1
19	0.1	21	0.15942817	17.2	13.2	0.1
19	0.3	17.5	0.15942817	17.2	13.2	0.1
19	1.3	17.2	0.15942817	17.2	13.2	0.1
19	3	15.4	0.15942817	17.2	13.2	0.1
19	5	13.7	0.15942817	17.2	13.2	0.1
19	7	12.1	0.15942817	17.2	13.2	0.1
19	9	10.6	0.15942817	17.2	13.2	0.1
19	11	5.8	0.15942817	17.2	13.2	0.1
19	13.2	0.001	0.15942817	17.2	13.2	0.1
20	0.1	19.1	0.138797723	15.8	13.4	0.1
20	0.3	17.2	0.138797723	15.8	13.4	0.1
20	1.3	15.8	0.138797723	15.8	13.4	0.1
20	3	14.6	0.138797723	15.8	13.4	0.1
20	5	12.7	0.138797723	15.8	13.4	0.1
20	7	11.5	0.138797723	15.8	13.4	0.1
20	9	9.2	0.138797723	15.8	13.4	0.1
20	11	5.7	0.138797723	15.8	13.4	0.1
20	13.4	0.001	0.138797723	15.8	13.4	0.1
21	0.1	26	0.223041454	19.1	15.1	0.1
21	0.3	21.5	0.223041454	19.1	15.1	0.1
21	1.3	19.1	0.223041454	19.1	15.1	0.1
21	3	17.4	0.223041454	19.1	15.1	0.1
21	5	16.1	0.223041454	19.1	15.1	0.1
21	7	13.6	0.223041454	19.1	15.1	0.1

21	9	12.4	0.223041454	19.1	15.1	0.1
21	11	9.2	0.223041454	19.1	15.1	0.1
21	13	4.5	0.223041454	19.1	15.1	0.1
21	15.1	0.001	0.223041454	19.1	15.1	0.1
22	0.1	18.1	0.116411559	15.4	12.4	0.1
22	0.3	16.4	0.116411559	15.4	12.4	0.1
22	1.3	15.4	0.116411559	15.4	12.4	0.1
22	3	13.7	0.116411559	15.4	12.4	0.1
22	5	11.7	0.116411559	15.4	12.4	0.1
22	7	9.7	0.116411559	15.4	12.4	0.1
22	9	7.4	0.116411559	15.4	12.4	0.1
22	11	4.4	0.116411559	15.4	12.4	0.1
22	12.4	0.001	0.116411559	15.4	12.4	0.1
23	0.1	11.5	0.027265568	8.8	8.7	0.1
23	0.3	9.8	0.027265568	8.8	8.7	0.1
23	1.3	8.8	0.027265568	8.8	8.7	0.1
23	3	7.9	0.027265568	8.8	8.7	0.1
23	5	6.7	0.027265568	8.8	8.7	0.1
23	8.7	0.001	0.027265568	8.8	8.7	0.1
24	0.1	25.8	0.17823873	21.7	13.1	0.1
24	0.3	23.7	0.17823873	21.7	13.1	0.1
24	1.3	21.7	0.17823873	21.7	13.1	0.1
24	3	17	0.17823873	21.7	13.1	0.1
24	5	14.2	0.17823873	21.7	13.1	0.1
24	7	9.9	0.17823873	21.7	13.1	0.1
24	9	5.9	0.17823873	21.7	13.1	0.1
24	13.1	0.001	0.17823873	21.7	13.1	0.1
25	0.1	23.7	0.172029844	18.4	12.9	0.1
25	0.3	19.5	0.172029844	18.4	12.9	0.1
25	1.3	18.4	0.172029844	18.4	12.9	0.1
25	3	17	0.172029844	18.4	12.9	0.1
25	5	14.1	0.172029844	18.4	12.9	0.1
25	7	12.2	0.172029844	18.4	12.9	0.1
25	9	8.6	0.172029844	18.4	12.9	0.1
25	11	4.9	0.172029844	18.4	12.9	0.1
25	12.9	0.001	0.172029844	18.4	12.9	0.1
26	0.1	15.6	0.08524802	12.5	12.3	0.1
26	0.3	14.4	0.08524802	12.5	12.3	0.1
26	1.3	12.5	0.08524802	12.5	12.3	0.1
26	3	12.4	0.08524802	12.5	12.3	0.1
26	5	11	0.08524802	12.5	12.3	0.1
26	7	8.2	0.08524802	12.5	12.3	0.1
26	9	6.5	0.08524802	12.5	12.3	0.1

26	12.3	0.001	0.08524802	12.5	12.3	0.1
27	0.1	19.3	0.094323374	16.4	9.6	0.1
27	0.3	18.1	0.094323374	16.4	9.6	0.1
27	1.3	16.4	0.094323374	16.4	9.6	0.1
27	3	13.7	0.094323374	16.4	9.6	0.1
27	5	9.7	0.094323374	16.4	9.6	0.1
27	7	5.8	0.094323374	16.4	9.6	0.1
27	9.6	0.001	0.094323374	16.4	9.6	0.1
28	0.1	12	0.032967088	9.5	6.7	0.1
28	0.3	11	0.032967088	9.5	6.7	0.1
28	1.3	9.5	0.032967088	9.5	6.7	0.1
28	3	8.5	0.032967088	9.5	6.7	0.1
28	5	8	0.032967088	9.5	6.7	0.1
28	6.7	0.001	0.032967088	9.5	6.7	0.1
29	0.1	23	0.171244603	19.6	12.6	0.1
29	0.3	20.6	0.171244603	19.6	12.6	0.1
29	1.3	19.6	0.171244603	19.6	12.6	0.1
29	3	17.2	0.171244603	19.6	12.6	0.1
29	5	14.7	0.171244603	19.6	12.6	0.1
29	7	11.7	0.171244603	19.6	12.6	0.1
29	9	7.3	0.171244603	19.6	12.6	0.1
29	12.6	0.001	0.171244603	19.6	12.6	0.1
30	0.1	22.7	0.135376647	19.9	11.5	0.1
30	0.3	20.8	0.135376647	19.9	11.5	0.1
30	1.3	19.9	0.135376647	19.9	11.5	0.1
30	3	16.4	0.135376647	19.9	11.5	0.1
30	5	9.8	0.135376647	19.9	11.5	0.1
30	7	7.2	0.135376647	19.9	11.5	0.1
30	9	6.1	0.135376647	19.9	11.5	0.1
30	11.5	0.001	0.135376647	19.9	11.5	0.1
31	0.1	20.1	0.114404356	17.5	9.6	0.1
31	0.3	19.8	0.114404356	17.5	9.6	0.1
31	1.3	17.5	0.114404356	17.5	9.6	0.1
31	3	14.3	0.114404356	17.5	9.6	0.1
31	5	11.7	0.114404356	17.5	9.6	0.1
31	7	8.7	0.114404356	17.5	9.6	0.1
31	9.6	0.001	0.114404356	17.5	9.6	0.1
32	0.1	16.3	0.068816587	12.4	9.2	0.1
32	0.3	14.6	0.068816587	12.4	9.2	0.1
32	1.3	12.4	0.068816587	12.4	9.2	0.1
32	3	11.7	0.068816587	12.4	9.2	0.1
32	5	9.8	0.068816587	12.4	9.2	0.1
32	7	6.7	0.068816587	12.4	9.2	0.1

32	9.2	0.001	0.068816587	12.4	9.2	0.1
33	0.1	34.3	0.490391892	27.7	15.7	0.1
33	0.3	30.5	0.490391892	27.7	15.7	0.1
33	1.3	27.7	0.490391892	27.7	15.7	0.1
33	3	25.6	0.490391892	27.7	15.7	0.1
33	5	23.6	0.490391892	27.7	15.7	0.1
33	7	20.9	0.490391892	27.7	15.7	0.1
33	9	18.5	0.490391892	27.7	15.7	0.1
33	11	14.7	0.490391892	27.7	15.7	0.1
33	13	10.5	0.490391892	27.7	15.7	0.1
33	15.7	0.001	0.490391892	27.7	15.7	0.1
34	0.1	22.2	0.209845548	17.8	13.5	0.1
34	0.3	19.1	0.209845548	17.8	13.5	0.1
34	1.3	17.8	0.209845548	17.8	13.5	0.1
34	3	17	0.209845548	17.8	13.5	0.1
34	5	15.7	0.209845548	17.8	13.5	0.1
34	7	13.8	0.209845548	17.8	13.5	0.1
34	9	11.9	0.209845548	17.8	13.5	0.1
34	11	10.4	0.209845548	17.8	13.5	0.1
34	13	5	0.209845548	17.8	13.5	0.1
34	13.5	0.001	0.209845548	17.8	13.5	0.1
35	0.1	17.1	0.104573488	13.9	12	0.1
35	0.3	15.8	0.104573488	13.9	12	0.1
35	1.3	13.9	0.104573488	13.9	12	0.1
35	3	13	0.104573488	13.9	12	0.1
35	5	11.6	0.104573488	13.9	12	0.1
35	7	10.3	0.104573488	13.9	12	0.1
35	9	8.4	0.104573488	13.9	12	0.1
35	12	0.001	0.104573488	13.9	12	0.1
36	0.1	29.6	0.230145224	22.3	12.8	0.1
36	0.3	25.8	0.230145224	22.3	12.8	0.1
36	1.3	22.3	0.230145224	22.3	12.8	0.1
36	3	20.1	0.230145224	22.3	12.8	0.1
36	5	17.2	0.230145224	22.3	12.8	0.1
36	7	12.8	0.230145224	22.3	12.8	0.1
36	9	5.3	0.230145224	22.3	12.8	0.1
36	12.8	0.001	0.230145224	22.3	12.8	0.1
37	0.1	25.2	0.163766513	18.6	12.4	0.1
37	0.3	20.6	0.163766513	18.6	12.4	0.1
37	1.3	18.6	0.163766513	18.6	12.4	0.1
37	3	16.8	0.163766513	18.6	12.4	0.1
37	5	14.4	0.163766513	18.6	12.4	0.1
37	7	11.3	0.163766513	18.6	12.4	0.1

37	9	7.4	0.163766513	18.6	12.4	0.1
37	12.4	0.001	0.163766513	18.6	12.4	0.1
38	0.1	17.2	0.065450371	13.4	10.9	0.1
38	0.3	15.6	0.065450371	13.4	10.9	0.1
38	1.3	13.4	0.065450371	13.4	10.9	0.1
38	3	10.8	0.065450371	13.4	10.9	0.1
38	5	8.5	0.065450371	13.4	10.9	0.1
38	7	5.3	0.065450371	13.4	10.9	0.1
38	10.9	0.001	0.065450371	13.4	10.9	0.1
39	0.1	42.2	1.061236831	39.4	22.7	0.1
39	0.3	40.3	1.061236831	39.4	22.7	0.1
39	1.3	39.4	1.061236831	39.4	22.7	0.1
39	3	35.3	1.061236831	39.4	22.7	0.1
39	5	32.1	1.061236831	39.4	22.7	0.1
39	7	29.9	1.061236831	39.4	22.7	0.1
39	9	25.7	1.061236831	39.4	22.7	0.1
39	11	22.2	1.061236831	39.4	22.7	0.1
39	13	19.6	1.061236831	39.4	22.7	0.1
39	15	16.7	1.061236831	39.4	22.7	0.1
39	17	11.5	1.061236831	39.4	22.7	0.1
39	19	6.5	1.061236831	39.4	22.7	0.1
39	22.7	0.001	1.061236831	39.4	22.7	0.1
40	0.1	28	0.217631514	21.5	13.6	0.1
40	0.3	23.9	0.217631514	21.5	13.6	0.1
40	1.3	21.5	0.217631514	21.5	13.6	0.1
40	3	18.7	0.217631514	21.5	13.6	0.1
40	5	17	0.217631514	21.5	13.6	0.1
40	7	13.2	0.217631514	21.5	13.6	0.1
40	9	9.2	0.217631514	21.5	13.6	0.1
40	13.6	0.001	0.217631514	21.5	13.6	0.1
41	0.1	20.3	0.178432724	18.7	13	0.1
41	0.3	19.5	0.178432724	18.7	13	0.1
41	1.3	18.7	0.178432724	18.7	13	0.1
41	3	16.9	0.178432724	18.7	13	0.1
41	5	14.8	0.178432724	18.7	13	0.1
41	7	12.5	0.178432724	18.7	13	0.1
41	9	9.5	0.178432724	18.7	13	0.1
41	11	5.6	0.178432724	18.7	13	0.1
41	13	0.001	0.178432724	18.7	13	0.1
42	0.1	14.2	0.070752908	13.2	10.7	0.1
42	0.3	13.4	0.070752908	13.2	10.7	0.1
42	1.3	13.2	0.070752908	13.2	10.7	0.1
42	3	12	0.070752908	13.2	10.7	0.1

42	5	9.9	0.070752908	13.2	10.7	0.1
42	7	7.5	0.070752908	13.2	10.7	0.1
42	10.7	0.001	0.070752908	13.2	10.7	0.1
43	0.1	46.5	0.690270266	35.5	17.1	0.1
43	0.3	40.5	0.690270266	35.5	17.1	0.1
43	1.3	35.5	0.690270266	35.5	17.1	0.1
43	3	31.7	0.690270266	35.5	17.1	0.1
43	5	27.3	0.690270266	35.5	17.1	0.1
43	7	24.7	0.690270266	35.5	17.1	0.1
43	9	18.1	0.690270266	35.5	17.1	0.1
43	11	13.6	0.690270266	35.5	17.1	0.1
43	13	10.1	0.690270266	35.5	17.1	0.1
43	17.1	0.001	0.690270266	35.5	17.1	0.1
44	0.1	36.5	0.574856517	30.8	17.5	0.1
44	0.3	34.1	0.574856517	30.8	17.5	0.1
44	1.3	30.8	0.574856517	30.8	17.5	0.1
44	3	27.5	0.574856517	30.8	17.5	0.1
44	5	25.2	0.574856517	30.8	17.5	0.1
44	7	21.9	0.574856517	30.8	17.5	0.1
44	9	19	0.574856517	30.8	17.5	0.1
44	11	15.1	0.574856517	30.8	17.5	0.1
44	13	10.7	0.574856517	30.8	17.5	0.1
44	15	6.9	0.574856517	30.8	17.5	0.1
44	17.5	0.001	0.574856517	30.8	17.5	0.1
45	0.1	34.1	0.493617091	27.9	16.7	0.1
45	0.3	31.1	0.493617091	27.9	16.7	0.1
45	1.3	27.9	0.493617091	27.9	16.7	0.1
45	3	25.7	0.493617091	27.9	16.7	0.1
45	5	23	0.493617091	27.9	16.7	0.1
45	7	20.4	0.493617091	27.9	16.7	0.1
45	9	18.4	0.493617091	27.9	16.7	0.1
45	11	14.8	0.493617091	27.9	16.7	0.1
45	13	9.5	0.493617091	27.9	16.7	0.1
45	15	4.8	0.493617091	27.9	16.7	0.1
45	16.7	0.001	0.493617091	27.9	16.7	0.1
46	0.1	12	0.030742055	10.1	7.6	0.1
46	0.3	11	0.030742055	10.1	7.6	0.1
46	1.3	10.1	0.030742055	10.1	7.6	0.1
46	3	8.3	0.030742055	10.1	7.6	0.1
46	5	5.1	0.030742055	10.1	7.6	0.1
46	7.6	0.001	0.030742055	10.1	7.6	0.1

h_i is the section height (m); D_i is the diameter at different heights (cm); v is the total tree volume (m^3); D is the diameter at breast height (1.3 m); H is the total tree height (m); h_0 is the average stump height (m).
