# Supplementary material for: Development of a Compatible Taper Function and Stand-Level Merchantable Volume Model for Chinese Fir Plantations
Source: PLoS One. 2016 Jan 22;11(1):e0147610. doi: 10.1371/journal.pone.0147610 (PMC4723312; doi:10.1371/journal.pone.0147610)
Supplement: S2 Table — (PDF) [file pone.0147610.s002.pdf]

Plot	H_d	D	H	G	N	d_g	V
1	11.7	16.6	9.8	9.8	442	16.8	48.6
2	11.3	13.4	9.2	11.7	796	13.7	58
3	11	14.1	9.6	10.7	651	14.4	54.3
4	8.8	11.6	8.2	2.8	265	11.7	12.5
5	13	15.1	10.5	17.1	916	15.4	94.2
6	13.8	15.6	10.7	29.9	1510	15.9	168
7	9.8	12.8	8.2	14.6	1093	13	62.1
8	11	14.6	9.9	13.7	796	14.8	69.7
9	15.4	25.9	14.8	15.2	286	26	101
10	16.1	17.3	11.6	29.2	1195	17.6	174.6
11	12.6	16.7	11.3	38	1712	16.8	213.8
12	8.2	12.8	8	5.9	442	13.1	24.9
13	11	14.7	9.7	17.1	973	15	87
14	7.2	7.4	6	10.2	2299	7.5	37.3
15	16.2	19	11.4	9.4	272	21	61.6
16	14.9	21.8	13	22.7	591	22.1	141.3
17	14.8	21.5	13.2	15.2	400	22	96.3
18	6.3	11.6	5.7	2.8	265	11.6	8.4
19	11.5	18.8	10	8.9	304	19.3	43.4
20	9.1	16.2	9	3.6	177	16.2	16.2
21	8.2	11.3	8.2	1.8	177	11.3	7.9
22	12.7	16.2	10.6	24.9	1157	16.6	136.4
23	13.2	16.2	10.8	23.2	1075	16.6	129.7
24	15.2	18	12.4	25.4	962	18.3	156.7
25	9.5	12.4	8.4	6.4	531	12.4	29
26	12.6	15.5	10.4	26.9	1390	15.7	143.8
27	13.8	16.1	10.4	30.9	1436	16.6	168.6
28	10	20.3	10	1	32	20.3	4.8
29	13.5	15.4	10.4	31.5	1630	15.7	171
30	11.9	23.2	11.6	5.4	127	23.3	28.5
31	9.8	12	8.4	17.6	1447	12.5	87.1
32	11.8	12.2	8.5	9.6	796	12.4	45.2
33	9.7	13.2	8.9	15.2	1093	13.3	71.4
34	9.1	12.1	8.1	13.4	1149	12.2	58.1
35	11.9	14.3	9.7	19.8	1181	14.6	102
36	11.5	14.1	9.7	30.9	1945	14.2	156.5
37	12.2	13.5	9.4	31.3	2122	13.7	158.1
38	12.9	16.1	10.8	20.5	980	16.3	112.6
39	13.9	16.6	10.8	19	842	16.9	106
40	11.3	13.6	9.5	25.5	1712	13.8	128.5
41	7	12.2	7	1	88	12.2	3.8
42	10.8	13.6	9.5	4.5	297	13.9	22.9

43	10.8	13.7	9.3	20.2	1326	13.9	100.4
44	12	14.6	9.9	14.5	828	14.9	75.3
45	14.8	17.7	11.6	35.8	1411	18	209.1
46	13.9	16.3	11.1	50.8	2377	16.5	286.4
47	13.7	14.8	10.3	31.3	1751	15.1	169.4
48	13.8	15.6	10.7	26.5	1333	15.9	147.4
49	14.4	17.4	10.9	15.3	576	18.4	90.1
50	14.9	17.2	11.7	27.1	1132	17.4	161.7
51	13.3	16.1	11	26.7	1277	16.3	150.8
52	15.8	18.7	12.7	16.2	576	18.9	101.4
53	11.6	14	9.8	18.6	1149	14.3	96.9
54	14.9	18.1	11.2	16.3	576	19	98
55	13.3	15.4	10.6	45.4	2345	15.7	251.6
56	13.1	16	10.9	48	2313	16.3	267.8
57	12.3	17.1	10.4	11.1	474	17.3	58.1
58	13.3	16.6	10.8	20.5	923	16.8	113.6
59	12.8	17.1	10.8	12.7	538	17.4	69.4
60	14.9	16.4	11	40.7	1846	16.8	232.8
61	14.2	16.8	11.1	47.8	2055	17.2	273.5
62	8.9	12.6	8.9	2.2	177	12.6	10.3
63	11.2	14.7	10.1	6.1	354	14.8	32.1
64	12.6	15.9	10.3	5.1	241	16.4	28.5
65	14.1	23.8	14.1	1.4	32	23.8	8.9
66	13.4	13.3	9.6	14.8	1061	13.3	75.9
67	9.2	12.5	8.7	25.3	2034	12.6	116.6
68	8.8	11.4	8.2	18.1	1768	11.4	80.5
69	13.5	13.3	9.5	38.8	2741	13.4	195.8
70	12.4	12.5	8.9	25.3	2009	12.7	123.7
71	9.9	13.7	8.6	3.8	209	15.2	19.8
72	10.5	13	9.1	19.6	1447	13.1	95.5
73	11.6	14.1	9.8	9.7	619	14.1	49.6
74	9.7	12	8.5	6.1	531	12.1	28.4

Hd is dominant height (m); D is the stand mean diameter (cm); H is the mean stand height (m); G is the stand basal area (m^2 ha^{-1}); N is the number of the trees per ha (trees ha^{-1}); d_g is quadratic mean diameter (cm); V is stand volume (m^3 ha^{-1}).
