# Supplementary material for: Development of a Compatible Taper Function and Stand-Level Merchantable Volume Model for Chinese Fir Plantations
Source: PLoS One. 2016 Jan 22;11(1):e0147610. doi: 10.1371/journal.pone.0147610 (PMC4723312; doi:10.1371/journal.pone.0147610)
Supplement: S1 Text — (PDF) [file pone.0147610.s003.pdf]

```
/*****************************************************************/
/*       PROGRAM TO FIT SIMULTANEOUSLY THE EQUATIONS SYSTEMS       */
/*        COMPOSED BY FANG ET AL. (2000) TAPER FUNCTION AND        */
/*           THE COMPATIBLE TOTAL TREE VOLUME EQUATION             */
/*                                                                 */
/*   INPUT VARIABLES:                                              */
/*                                                                 */
/*   d = top diameter with bark (cm) at height h (cm)              */
/*   D = the diameter at breast height (1.3 m aboveground, cm)     */
/*   H = total tree height (m)                                     */
/*   h = height aboveground (m) to top diameter d (m)              */
/*   hst = stump height (m)                                        */
/*   v = merchantable volume with bark from the stump to           */
/*       the height h (m^3)                                        */
/*   V = total tree volume (m^3)                                   */
/*   k = p/40000, a metric constant for converting from the        */
/*       diameter squared in cm2 to cross-section area in m2       */
/*   q = h/H                                                       */
/*   dataNumber = number of diameter (d) observations by tree      */
/*   tree       = tree number                                      */
/*   power      = weighting factor previously obtained using the   */
/*                approach proposed by Harvey (1976).              */
/*                                                                 */
/*   OUTPUT PARAMETERS:                                            */
/*                                                                 */
/*   a0 a1 a2 = parameters common to the taper function and the    */
/*              total tree volume                                  */
/*   b1 b2 b3 = form factors of sections 1 to 3                    */
/*   p1 p2    = relative height from the ground level where the    */
/*              two inflection points assumed in the model occur   */
/*   rhoi     = i-order continuous autoregressive parameters       */
/*                                                                 */
/*   References: Fang ZX, Borders BE, Bailey RL (2000) Compatible  */
/*               volume-taper models for loblolly and slash pine   */
/*               based on a system with segmented-stem form        */
/*               factors. Forest Science 46: 1-12.                 */
/*                                                                 */
/*               Harvey AC (1976) Estimating regression models     */
/*               with multiplicative heteroscedasticity.           */
/*               Econometrica 44: 461-465.                         */
/*                                                                 */
/*****************************************************************/

/*****************************************************************/
/* MACRO TO CREATE THE MODIFIED CONTINUOUS AUTOREGRESIVE STRUCTURE */
/*          IN THE CODE A mCAR(3) STRUCTURE WAS USED               */
/*****************************************************************/

%MACRO DATA_AUTOCORR;
     DATA taperg;
     SET taper;       *--- taper is the name of the original file;

          %DO i=1 %TO 3 %BY 1;
          dist&i=DIF&i(hi);
               IF tree NE LAG&i(tree)
               THEN DO dist&i=0; d&i=0;
               END;
          ELSE d&i=1;
```

```
            %END;
      RUN;  QUIT;
%MEND;

%DATA_AUTOCORR;
RUN; QUIT;

/*************************************************************** */
/*          SIMULTANEOUS FITTING WITH A mCAR(3)STRUCTURE         */
/*************************************************************** */

proc model data = taperg;

      parms a0 a1 a2 b1 b2 b3 p1 p2 rho1 rho2 rho3;

*--- VARIABLES DEFINITION;

      k  = ( 3.1415926/40000);
      z  = h/H;
      I1 = (max((z-p1),0)/(z-p1))*(max((p2-z),0)/(p2-z));
      I2 =  max((Z-p2),0)/(Z-p2);
      b  = (b1**(1-(I1+I2)))*(b2**I1)*(b3**I2);
      f1 = (1-p1)**(((b2-b1)*k)/(b1*b2));
      f2 = (1-p2)**(((b3-b2)*k)/(b2*b3));
      t2 = (1-p2)**(k/b2);
      t1 = (1-p1)**(k/b1);
      p0 = (htoc/H);
      t0 = (1-p0)**(k/b1);
      c1 = sqrt((a0*(d**a1)*(h**(a2-(k/b1))))/(b1*(t0-t1)
           +b2*(t1-f1*t2)+b3*f1*t2));

*--- TOTAL TREE VOLUME EQUATION;

      v  = a0*(d**a1)*(h**a2);

*--- WEIGHTING FACTOR FOR HETEROCEDASTICITY AND TO ACCOUNT FOR THE;
*--- DIFFERENT NUMBER OF OBSERVATIONS;

      resid.v = resid.v*sqrt(1/(dataNumber*(d**2*h)**power));

*--- TAPER FUNCTION;

      d = c1*sqrt(H**((k-b1)/b1)*((1-z)**((k-b)/b))*(f1**(I1+I2))
           *(f2**I2))

*--- MODIFIED CONTINUOS AUTOREGRESIVE STRCUTURE OR ORDER 3 -mCAR(3)-;

      -d1*rho1**dist1*ZLAG1(RESID.di)-d2*rho2**dist2*ZLAG2(RESID.di)
      -d3*rho3**dist3*ZLAG3(RESID.di);

*--- SIMULTANEOUS FIITING AND INITIAL PARAMETERS;

      fit di v
       start = (a0 = 0.000014 a1 = 1.62 a2 = 0.95 b1 = 0.000017
                b2 = 0.000032 b3 = 0.000027 p1 = 0.13 p2 = 0.58
                rho1 = 0.4 rho2 = 0.6 rho3 = 0.55)/ itsur;
run;
quit;
```

```
/*****************************************************************************/
/*    PROGRAM TO FIT A STAND-LEVEL MERCHANTABLE VOLUME EQUATION     */
/*                                                                  */
/*   INPUT VARIABLES:                                               */
/*                                                                  */
/*   Vol    = stand-level merchantable volume (m^3 ha^-1)           */
/*   G      = stand basal area(m^2 ha^-1)                           */
/*   H      = stand dominant height (m), the average height of the */
/*            100 largest trees per ha                              */
/*   dg     = quadratic mean diameter (cm)                          */
/*   di     = top diameter with bark (cm)                           */
/*   power  = weighting factor previously obtained using the        */
/*            approach proposed by Harvey (1976).                   */
/*                                                                  */
/*   OUTPUT PARAMETERS:                                             */
/*                                                                  */
/*   c0 c1 c2 = parameters of the total stand volume equation       */
/*   c3 c4 c5 = parameters of the stand volume ratio equation       */
/*                                                                  */
/*   Reference: Harvey AC (1976) Estimating regression models with */
/*              multiplicative heteroscedasticity.                  */
/*              Econometrica 44: 461-465.                           */
/*****************************************************************************/

/*****************************************************************************/
/*   FITTING WITH A WEIGHTING FACTOR TO CORRECT HETEROCEDASTICITY   */
/*****************************************************************************/

proc model
data = standvolume; *--- standvolume is the name of the original file;

*--- STAND-LEVEL MERCHANTABLE VOLUME EQUATION;

      Vol = c0*(G**c1)*(H**c2)*exp(c3*di**c4*dg**c5);

*--- WEIGHTING FACTOR;

      resid.Vol = resid.Vol*sqrt(1/((G*H)**power));

*-- FIITING AND INITIAL PARAMETERS;

      fit Vol
       start = (c0 = 1.3 c1 = 0.9 c2 = 0.6 c3 = -0.7 c4 = 4 c5 = -4);
run;
quit;
```
